# Temporomandibular Joint Dislocation in Patients With Parkinsonism: A Report of Two Cases

**DOI:** 10.7759/cureus.81345

**Published:** 2025-03-28

**Authors:** Koji Hayashi, Akiho Maeda, Asuka Suzuki, Yuka Nakaya, Mamiko Sato, Naoko Takaku, Toyoaki Miura, Yasutaka Kobayashi

**Affiliations:** 1 Department of Rehabilitation Medicine, Fukui General Hospital, Fukui, JPN; 2 Department of Neurology, University of Fukui, Fukui, JPN; 3 Graduate School of Health Science, Fukui Health Science University, Fukui, JPN

**Keywords:** parkinsonism, parkinson-plus syndrome, parkinson's disease, temporomandibular joint dislocation, temporomandibular joint disorder

## Abstract

This report presents two cases of temporomandibular joint (TMJ) dislocation in patients with L-dopa-responsive Parkinsonism, highlighting an underrecognized complication of the disease. Both patients, classified as Hoehn-Yahr stage IV, exhibited typical Parkinsonism symptoms, such as tremors, rigidity, and gait disturbances. TMJ dislocations occurred in both patients during events involving wide mouth opening, one during breakfast and the other while yawning. The underlying pathophysiology remains unclear but may be related to Parkinsonism-associated muscle rigidity or involuntary orofacial movements. Manual reduction was performed in both cases, though difficulties were encountered due to increased muscle tone. These cases underscore the importance of early recognition and proactive management of TMJ dysfunction in Parkinsonism patients, especially in cases of advanced disease. Future studies should explore preventative strategies and optimal management techniques to improve patient outcomes. Accumulating data from similar cases will be essential for developing more effective management strategies for TMJ dysfunction in patients with Parkinsonism.

## Introduction

Temporomandibular joint (TMJ) dislocation, also known as mandibular dislocation, involves the displacement of the mandibular condyle from the glenoid fossa of the temporal bone [1]. Dislocations can occur in various directions, including anterior, posterior, superior, and lateral [1]. Anterior TMJ dislocations, the most common type, occur when the mandibular condyle displaces forward past the articular eminence of the temporal bone [1]. This displacement can be caused by excessive elevation of the mandible by the temporalis and masseter muscles, particularly before the lateral pterygoid muscle relaxes [1]. Such dislocations frequently occur due to extreme mouth opening during activities like eating, yawning, laughing, singing, kissing, vomiting, or dental procedures [2-4]. Other potential causes include trauma, dystonic reactions to medications, seizures, tetanus infection, and iatrogenic events during anesthesia or endoscopy [5-9]. TMJ dislocations can occur unilaterally or bilaterally, requiring a careful assessment of mandibular symmetry by healthcare providers [1].

Understanding risk factors is crucial for comprehending potential causes and evaluating the risk of recurrent TMJ dislocations. Significant risk factors include a prior history of dislocation, structural or anatomical abnormalities, connective tissue disorders compromising stability (such as Marfan syndrome, Ehlers-Danlos syndrome, muscular dystrophy, and orofacial dystrophy), neurodegenerative or neurodysfunctional conditions (including Huntington's disease, multiple sclerosis, and epilepsy), increasing age, and alterations in dental structure [1]. While a few case reports link TMJ dislocation to Parkinson's disease [10, 11], these reports are scarce and mostly from older literature.

It is important to note that the prognosis of TMJ dislocation is generally favorable, particularly for anterior dislocations [1]. However, complications, such as increased recurrence risk even after proper management, may adversely affect patients' quality of life [1]. Persistent TMJ dysfunction can lead to chronic pain, difficulties with eating and speaking, and psychological distress [1]. Thus, understanding the relationship between TMJ dislocation and conditions like Parkinsonism not only enhances clinical management strategies but also aims to improve overall patient outcomes and quality of life.

This report presents two cases of TMJ dislocation associated with Parkinsonism and discusses potential etiologies based on previous literature.

## Case presentation

Case 1

**Figure 1:**
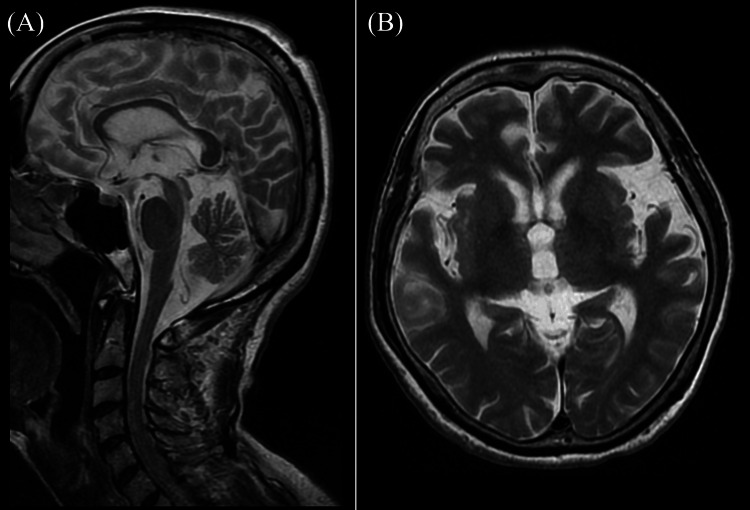
Brain magnetic resonance imaging (MRI) findings. (A, B) Brain MRI revealed no significant abnormalities, including brainstem atrophy or basal ganglia region abnormalities.

A 72-year-old woman with a history of breast cancer, diagnosed at age 71, developed left-hand clumsiness and a tendency to fall backward. These symptoms gradually worsened, prompting her to visit her previous hospital. Neurological findings included masked facies, a positive Myerson's sign, saccadic eye movements without any restriction in vertical eye movements, left-dominant resting, postural and action tremors, and cogwheel rigidity, mainly in the distal muscles. She exhibited a small-step gait, a significant tendency to fall backward, often collapsing like a log, and mild constipation. Dizziness was not noticeable. A brain MRI showed no significant abnormalities, including brainstem atrophy or basal ganglia region abnormalities (Figure 1).

**Figure 2:**
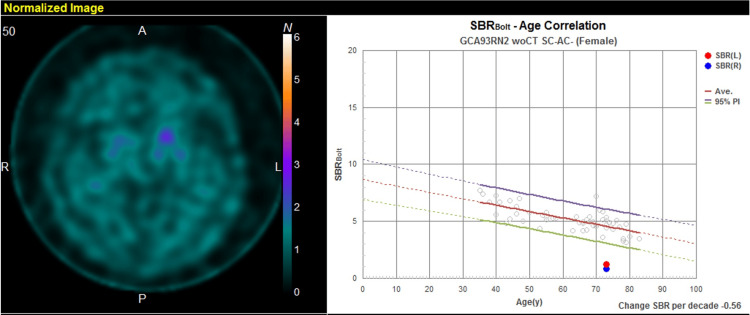
Dopamine transporter single-photon emission computed tomography (DaT-SPECT) with 123I-Ioflupane findings. DaT-SPECT demonstrated decreased accumulation predominantly on the right side (striatal binding ratio {SBR}: right 1.41, left 2.34).

Dopamine transporter single-photon emission computed tomography (DaT-SPECT) indicated decreased accumulation predominantly on the right side (striatal binding ratio: right 1.41, left 2.34) (Figure 2).

**Figure 3:**
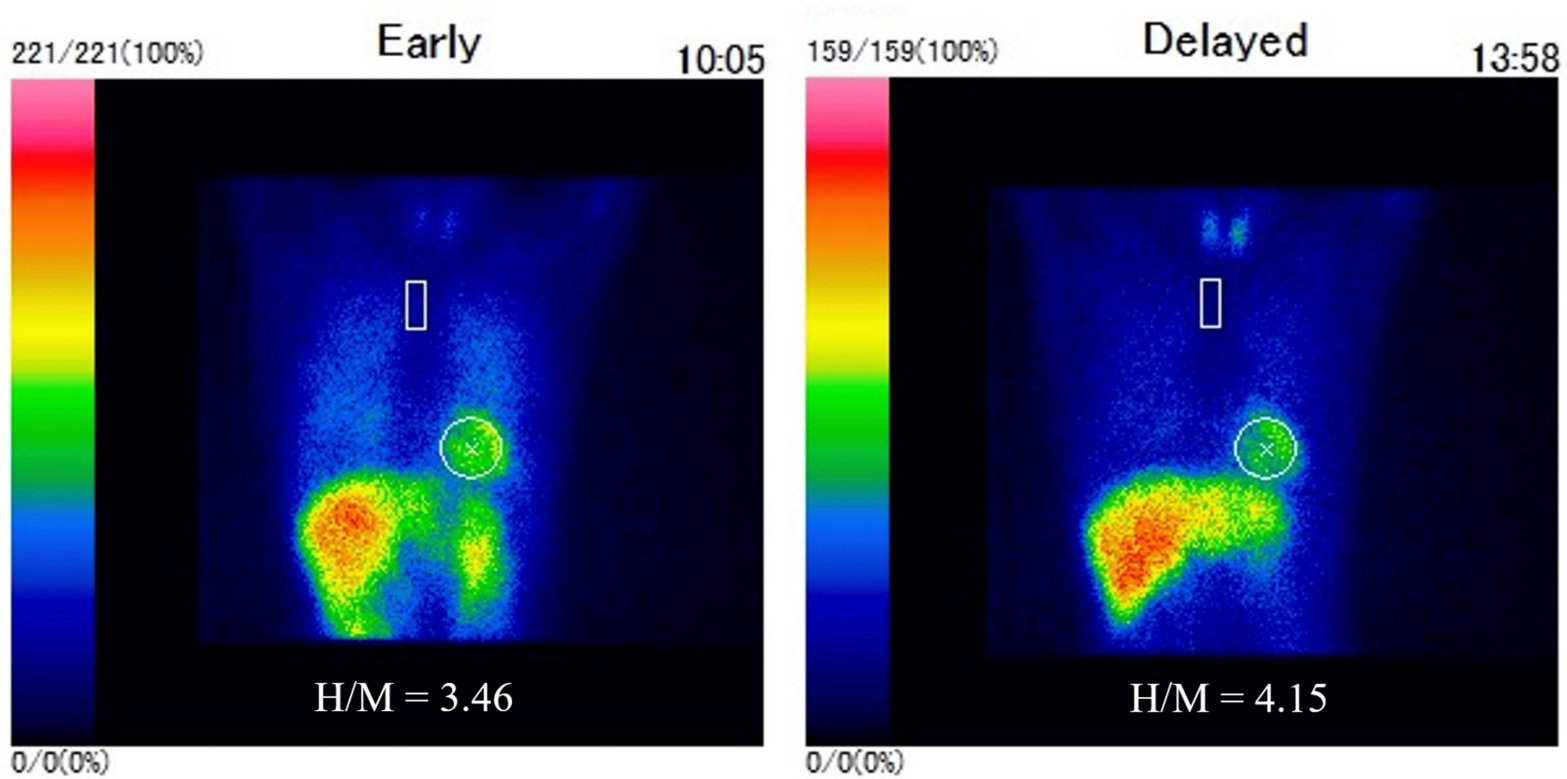
123I-metaiodobenzylguanidine (MIBG) myocardial scintigraphy findings. MIBG myocardial scintigraphy showed normal accumulation of the nuclide in the heart with a heart/mediastinum (H/M) ratio of 3.46/4.15 (early/delayed).

A ^123^I-metaiodobenzylguanidine (MIBG) myocardial scintigraphy was evaluated as normal (Figure 3). Parkinson's disease (PD) or progressive supranuclear palsy (PSP) were suspected; however, due to notable improvement in motor symptoms following the L-dopa challenge test, she was ultimately diagnosed with PD (Hoehn and Yahr {HY} stage 3). She was referred to our hospital for rehabilitation and medical treatment, where she continued to receive treatment with L-dopa at a dose of 200 mg/day.

At around age 73, the frequency of her falls increased further, occurring about twice a day, leading her to use a wheelchair (HY stage 4). The dosage of L-dopa was increased to 250 mg, and an 8 mg transdermal patch of ropinirole was added, resulting in mild improvement in her tendency to fall and walking difficulties. The ropinirole patch dosage was gradually increased as symptoms worsened, with improvement seen after each increase. By age 74, the dosage had reached 32 mg. At age 75, she contracted COVID-19 and received encitrelvir. Following infection, she became unable to walk or eat, even with assistance. Her muscle tone was exacerbated compared to before the COVID-19 infection, although jaw tremors were not noted. She was admitted to our hospital for rehabilitation. She underwent rehabilitation after admission, and her physical functions gradually returned to their previous level. However, she remained dependent on assistance for eating, and swallowing rehabilitation was the main focus. Additionally, her head was tilted back in a chin-upward posture whenever she sat on her bed. On the 11th day after admission, while eating breakfast with assistance, she suffered a TMJ dislocation while opening her mouth wide. Manual reduction was immediately performed but with some difficulty. Specifically, achieving manual reduction required a significant amount of force and took a considerable amount of time. She wore a mandibular fixation device for several days, and no TMJ dislocation was observed during her subsequent hospitalization.

Case 2

A 77-year-old woman with a history of post-operative gastric cancer, osteoporosis, vertebral compression fractures, and an adrenal tumor visited our hospital for dizziness, right lateropulsion, and gait disturbances. She had no history of smoking, alcohol consumption, diabetes, hypertension, or dyslipidemia. Neurological examination revealed resting and postural tremors in the left hand, along with cogwheel rigidity in the left limbs. Jaw tremor was not noted. The finger-to-nose test showed no dysmetria but revealed a left action tremor. The hand pronation test demonstrated significant awkwardness in her left-hand dominance, disrupting the rhythm. She was unable to move or walk independently and was admitted for peripheral vertigo, receiving treatment with anti-dizziness medication. Throughout her hospitalization, she exhibited a backward head tilt whenever she sat on her bed. On day five after admission, she was observed to have bilateral TMJ dislocations while yawning. Immediately following the dislocation, a dentist performed manual reduction, but with some difficulty. Similar to the patient in Case 1, this process required substantial force and considerable time to complete. She was discharged from the hospital on day 14. After discharge, she experienced recurrent dizziness on two occasions, and physicians noted parkinsonism during each of her visits to our hospital.

**Figure 4:**
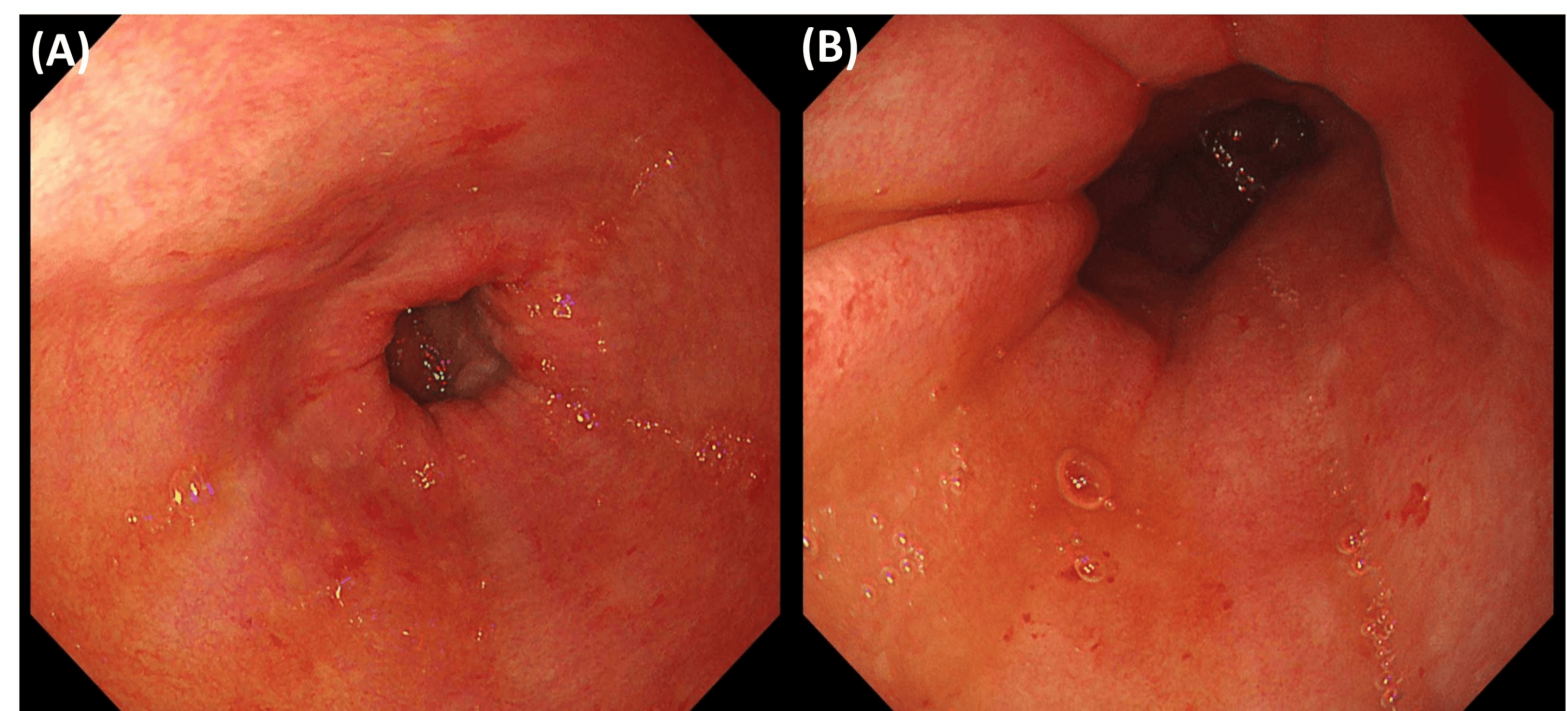
Upper gastrointestinal endoscopy findings. (A & B) Endoscopy revealed both redness and oozing bleeding in the stomach. The cause of the bleeding could not be identified.

Five months after the initial admission, she experienced hematemesis, leading to re-admission. She underwent upper gastrointestinal endoscopy, but the cause of the bleeding could not be identified (Figure 4). However, she remained unable to move or walk independently after re-admission. On day 17 after readmission, she experienced recurrent bilateral TMJ dislocation while yawning. Manual reduction was performed, but it was somewhat difficult, similar to the previous episode. She was instructed to avoid opening her mouth wide. Additionally, an L-dopa challenge test was initiated on day 17. The results were dramatic, allowing her to self-transfer to a wheelchair and resulting in a reduction of her resting tremor. Despite the absence of other nuclear testing findings, including MIBG myocardial scintigraphy and DaT-SPECT, PD was clinically suspected (HY stage IV). She was discharged on a maintenance dose of 300 mg of L-dopa. The anti-Parkinson's medications remained effective even after discharge, and her activities of daily living remained relatively stable.

## Discussion

We describe two cases of Parkinsonism presenting with TMJ dislocation. Both cases were L-dopa-responsive Parkinsonism, although a definitive diagnosis of PD was not established, classified as HY stage IV, and the dislocations occurred during hospitalization. Regarding the TMJ dislocation, both cases were first-time episodes; however, Case 2 experienced two episodes of dislocation within a short period. Both cases were treated with manual reduction, although some difficulty was encountered. It required significant force and took a considerable amount of time to achieve. These cases suggest that Parkinsonism may cause TMJ dislocation and demonstrate that the clinical symptoms of Parkinsonism can have a significant impact on TMJ.

Baram et al. reported on orofacial function and TMJ disorders in PD [12]. The authors recruited 20 individuals with PD and 20 age- and gender-matched controls, analyzing both objective assessments and subjective reports. The study indicated that PD patients had poorer orofacial function and exhibited significantly greater limitations in jaw mobility and function compared to the control group. Additionally, Choi et al. reported that PD is associated with a significantly higher risk of TMJ disorders, with an adjusted odds ratio of 1.43 for TMJ disorders compared to non-PD individuals using data from the Korean National Health Insurance Service Health Screening Cohort [13]. The authors believe that TMJ disorders in PD patients are caused by factors such as teeth grinding, jaw tremors, and muscle stiffness. Thus, these studies suggest that the clinical manifestations of PD have a significant impact on TMJ, not limited to jaw dislocation.

As far as we know, there are some previous reports regarding TMJ dislocation in Parkinsonism. Merrill first reported habitual TMJ dislocation in Parkinsonism in 1968 [10]. The case involved a 56-year-old patient with post-influenza Parkinsonism at age 14, characterized by tremors in the head, neck, perioral region, and limbs, as well as hypersalivation and ocular spasms. The authors hypothesize that the mechanism of habitual TMJ dislocation is due to the sustained tremors in the perioral region over 42 years, leading to deformation of the articular cartilage and condyles, which in turn caused stretching of the joint ligaments. Sayama et al. reported two cases of PD with habitual TMJ dislocation in 1999 [11]. Both cases had a history of neuroleptic malignant syndrome and were classified as HY stage V, exhibiting marked retroflexion and neck rigidity. Notably, both cases presented with difficulty in manual reduction of the TMJ dislocation. The authors speculated that increased tension in the suprahyoid muscles, which are involved in mouth opening, during neck hyperextension might have contributed to the TMJ dislocation. Furthermore, there is a report of a high dose of oral haloperidol leading to bilateral TMJ dislocation following a suicide attempt [14]. It is well-known that a side effect of haloperidol is the occurrence of extrapyramidal symptoms, including parkinsonism. The authors attributed the etiology to oromandibular dystonia related to haloperidol, which subsequently resulted in TMJ dislocation.

A retrospective questionnaire survey regarding TMJ dislocation was conducted in 2012 in Japan, targeting 262 facilities, including training facilities of the Japanese Society of Oral and Maxillofacial Surgeons [15]. Among 2461 cases with TMJ dislocation, 879 had no previous medical history. Regarding neurological disorders, 303 cases had cerebrovascular disease, 235 had dementia, and 133 cases had PD. Given that the prevalence of PD is estimated to be 1-2 per 1000 of the population [16], significantly lower than that of cerebrovascular disease and dementia, the proportion of PD among TMJ dislocation cases is remarkably high. These results suggest that TMJ dislocation in individuals with PD is not uncommon and may be underreported in clinical practice.

In our cases, although a definitive diagnosis of PD was not confirmed, both patients exhibited advanced stages of L-dopa-responsive parkinsonism, classified as HY stage IV. This was characterized by an involuntary posture with their heads tilted backward and mouths open, likely due to the high muscle tone throughout their bodies, including the face and trunk, associated with this advanced stage. We speculate that the TMJ dislocation in these cases may have been caused by a combination of factors: increased muscle rigidity in the neck and jaw, a consequence of advanced Parkinsonism, and the wide mouth opening associated with actions like yawning or feeding. This combination likely placed significant stress on the TMJ. The stiffness of these muscles may have also contributed to the difficulty encountered during manual reduction, consistent with previous reports. Interestingly, despite prior documentation of tremors in similar cases, neither patient exhibited jaw tremors. However, mandibular tremors are a common phenomenon in advanced Parkinsonism and are known to strain the TMJ, suggesting they could also contribute to TMJ dislocation. Taken together, our report and previous studies suggest that TMJ dislocation may be a common sign of advanced Parkinsonism. Further research and accumulation of similar cases are needed to elucidate the underlying mechanisms of TMJ dislocation in Parkinsonism.

## Conclusions

We present two cases of TMJ dislocation related to Parkinsonism. Previous large-scale surveys in Japan have indicated that TMJ dislocation associated with Parkinsonism is relatively common, suggesting it may be underreported in clinical practice. The potential causes of TMJ dislocation in patients with Parkinsonism may include increased muscle tone surrounding the TMJ during advanced stages of the disease and the wide-mouth opening associated with activities such as yawning or feeding.

Our cases highlight the importance of recognizing TMJ dislocation as a potential complication of advanced Parkinsonism and the need for further research to better understand the underlying mechanisms involved. Accumulating data from similar cases will be essential to develop more effective management strategies for TMJ dysfunction in patients with Parkinsonism.
